# Self-Resolution of a Draining Sinus Tract in a Patient with Chronic Periprosthetic Hip Infection

**DOI:** 10.1155/2018/8657562

**Published:** 2018-02-19

**Authors:** Trevor J. Shelton, Alton W. Skaggs, Gavin C. Pereira

**Affiliations:** ^1^Department of Orthopaedics, University of California, Davis, 4860 Y Street, Suite 3800, Sacramento, CA 95817, USA; ^2^School of Medicine, University of California, Davis, Sacramento, CA 95817, USA

## Abstract

We report a novel case of a patient who had a draining sinus soon after a total hip arthroplasty that spontaneously resolved. The patient voluntarily discontinued antibiotic suppressive therapy (AST) after 10 years of treatment and paradoxically experienced full resolution of signs of chronic prosthetic joint infection (PJI), including recovery of his left-sided draining sinus tract. Now 8 years after discontinuing AST, the patient has no pain, good function, and no major or minor criteria of joint infection according to the Musculoskeletal Infection Society (MSIS) workgroup. The authors have not identified literature describing a similar resolution of draining sinus tracts from around a prosthetic joint after discontinuing AST. Despite the resolution of this patient's sinus tract, the authors do not advocate for discontinuing AST in patients with a draining sinus tract. However, in spite of the fact that the MSIS consensus statement suggests that a draining sinus is a sure sign of PJI and that the assumption is that the infection will not go away until explant, this case was different.

## 1. Introduction

Total hip arthroplasty (THA) is a routine and highly successful procedure that provides patients with reduced pain, increased joint mobility, and improved vitality. However, despite aggressive aseptic protocol surrounding surgical procedures, periprosthetic joint infection (PJI) remains a leading cause of surgical revision and is the most serious complication surrounding THA [1]. In total, it has been reported that acute or chronic infection occurs in 0.3%–1.7% of patients after undergoing THA [2–4]. According to the Musculoskeletal Infection Society (MSIS) workgroup, there are two major criteria for diagnosing a PJI: (1) a sinus tract communicating with the prosthesis, or (2) a pathogen is isolated by culture from at least two separate tissue or fluid samples obtained from the prosthetic joint [5].

A relatively small number of organisms are required to contaminate a newly placed prosthesis. Possible origins of the offending pathogens include host skin microbiota, iatrogenic contamination, or hematogenous spread. Colonization of the implant is assisted by the formation of a biofilm, which protects microorganisms both from the host immune system and antimicrobial agents, making treatment particularly difficult [6].

Acute infections are most commonly caused by virulent bacterial strains and occur within the first three months of recovery—often accompanied by warmth, pain, and erythema at the site of infection, along with constitutional symptoms. The primary treatment of such is debridement, antibiotics, and implant retention (DAIR), with a reported success rate between 60 and 80% [7]. There are many risk factors associated with the preoperative, perioperative, and postoperative course which are associated with developing PJI (Table 1) [4, 8–10]. In contrast with acute PJI, chronic infection is often caused by less virulent bacterial strains and may run a more indolent course where symptoms may not manifest for months or years postoperatively [9]. In addition, erythema, swelling, and constitutional symptoms are generally absent in chronic infection. Patients will likely present with pain and joint instability alone. Progression of infection may lead to prosthetic loosening, muscle/soft tissue abscess, and eventually the development of a draining sinus tract [9].

In order to thoroughly clear infection, surgical revision of the prosthesis is generally required. However, in individuals with a high risk for revision failure due to advanced age, immunosuppressed state, malignancy, or other comorbidities, antibiotic suppressive therapy (AST) is recommended. While unlikely to resolve an existing infection, the success rate of AST is reported to be between 63% and 86% and is therefore considered a useful treatment modality in patients unable to tolerate surgical revision. Unfortunately, long-term antibiotic therapy is associated with significant side effects including gastrointestinal distress and antibiotic resistance, which can be quite detrimental to a patient's well-being and interfere with medication compliance.

We report a novel case in which a patient voluntarily discontinued AST after 10 years of treatment, and paradoxically experienced full resolution of all signs of chronic PJI including recovery of his left-sided draining sinus tract and has had no signs of infection over the last 10 years.

## 2. Case Presentation

**Figure 1:**
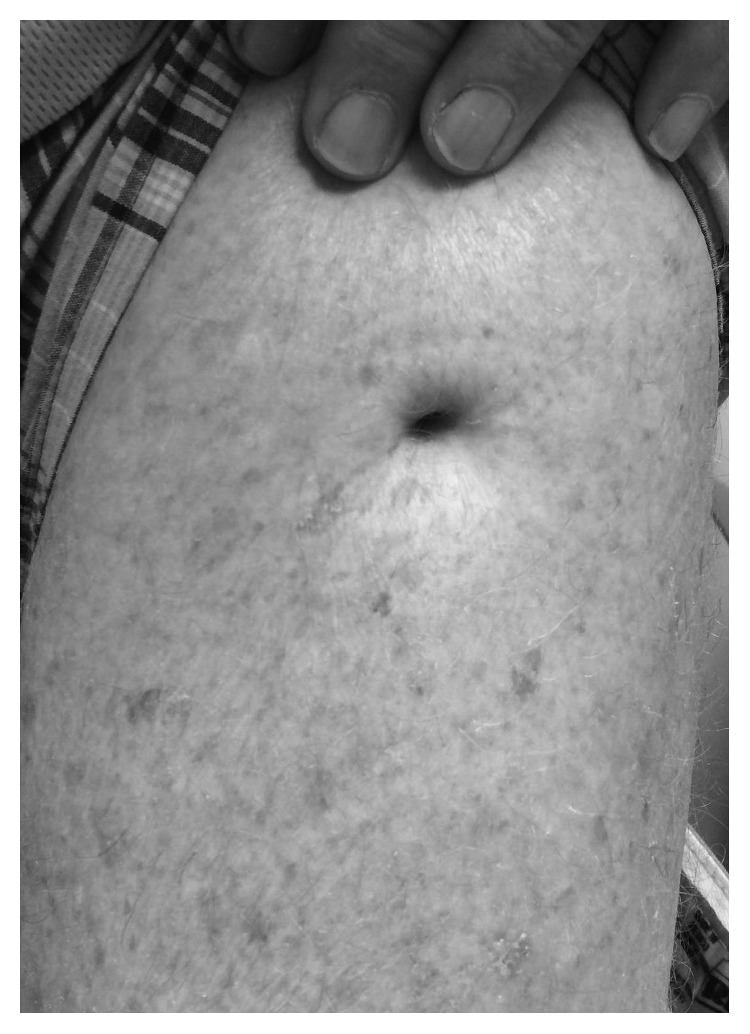
Dry sinus tract of a chronic periprosthetic left hip infection that began 20 years ago. Tract has been dry for the last 12 years.

We report the case of a 73-year-old male with bilateral THAs performed simultaneously at a different institution in 1995 using Biomet Bi-Metric stem and Ringloc cup through a posterior approach. Preoperatively, the patient had debilitating bilateral hip pain (right worse than left), and the patient reported shortening of his right leg compared to his left. The surgery was performed without complications, and the patient reported no problems with his THAs for two years with the exception that his right leg was shorter than his left. One year after surgery, the patient had a good Harris Hip Score of 87. Two years after his bilateral THAs, the patient reports developing a draining sinus tract of his left thigh (Figure 1) and bilateral periprosthetic infections which were not confirmed with aspiration. The patient did not want to have a revision surgery and was placed on chronic AST which was managed without the aide of infectious disease or identification of the microorganism. The patient had no risk factors for PJI.

**Figure 2:**
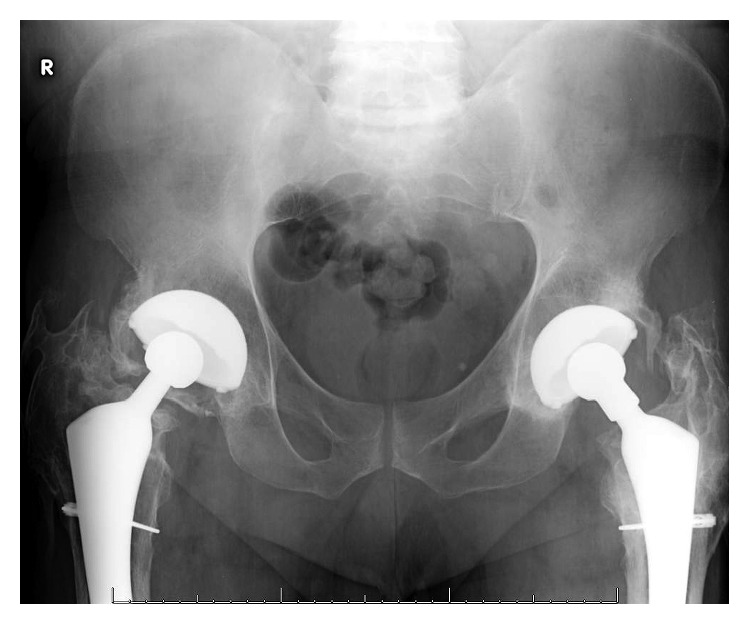
Plain radiograph of pelvis in a patient with history of bilateral periprosthetic infections 9 years following bilateral total hip arthroplasties. X-ray demonstrates good fixation of implant without loosening, some eccentric wear on the right polyethylene liner, no periosteal reaction. There is bilateral Brooker III/IV heterotopic ossification.

In 2004, the patient was first seen in clinic and reported doing well with no hip pain but had some decreased hip range of motion and noted to have Brooker III/IV heterotopic ossification around both hips on X-ray (Figure 2). He denied any systemic symptoms but was still having some drainage from the sinus of his left thigh. He was encouraged to continue his antibiotics, which at the time was amoxicillin.

By his next visit in 2005, the patient had started to wean himself off his amoxicillin as there had not been any drainage from his sinus tract in several months. A discussion took place with the patient in which it was explained to him that there were risks associated with discontinuing his antibiotics and that they had been beneficial in suppressing the patient's hip infection to that point in time. The patient expressed understanding and said he would continue to monitor his symptoms but was going to continue to wean himself off the antibiotics and would be sure to follow up if there were any complications.

**Figure 3:**
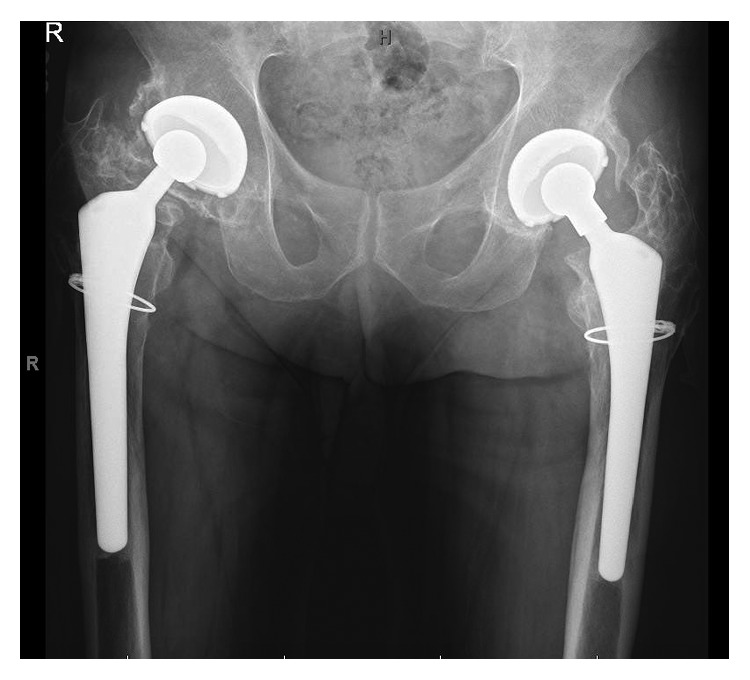
Plain radiograph of pelvis in a patient with history of bilateral periprosthetic infections 20 years following bilateral total hip arthroplasties. X-ray demonstrates good fixation of implant without loosening, some eccentric wear on the right polyethylene liner, no periosteal reaction. There is bilateral Brooker III/IV heterotopic ossification.

The patient returned in 2015 as he wanted an update about his hip replacements. During this visit, the patient stated he did not have any pain in his hips and has had no drainage from his thigh in the previous 8 years. He has not had any fevers, chills, or systemic signs of infection. His only complaint was reduced range of motion in his hips. On physical exam, the patient had a healed up and dry sinus tract opening over the anterior aspect of his proximal left thigh, no tenderness to palpation over his hips, and no fluctuance or erythema. He had 10° of internal rotation, 15° of external rotation, and 40–50° of hip flexion bilaterally with no pain with log roll. Plain radiographs showed well-fixed bilateral total hip replacements. On the contralateral right side, there was evidence of some eccentric wear of the polyethylene liner and only minimal wear on the left side. There was no evidence of periosteal reaction, osteolysis, or cloacal openings seen anywhere along the left proximal femur. There was continued presence of Brooker III/IV heterotopic ossification around both hips without subsidence of his left hip compared to his X-rays in 2014 (Figure 3). Now, twelve years after stopping AST therapy, the patient continues to have no signs of infection or loosening or wear in his hip and reports a good Harris Hip Score of 84.

## 3. Discussion

THA is a highly successful and common procedure that provides patients with vastly improved quality of life. Recent reports suggest that the survival rate of THA is upwards of 95% at 10-year follow-up in patients over 75 years of age [1]. However, behind aseptic loosening and general joint instability, PJI remains the third leading cause of complication after total hip arthroplasty [1]. It is agreed upon that the presence of a sinus tract that communicates with the prosthesis is definitive evidence of PJI [5, 11]. The most important finding of this case demonstrates the spontaneous resolution of a draining sinus tract after a patient weaned himself off AST and has had no signs of infection over the last 10 years. And with no major or minor criteria to suggest a PJI using the MSIS criteria [5], no pain, good function, and no radiological signs of infection, the probability is that he does not have an ongoing infection.

Surgical revision remains the gold standard of care in patients who develop PJI following THA and is the only route to reliably clear an underlying infection. Revision involves removal of the prosthetic and associated cement, followed by aggressive debridement, parenteral antibiotics, and placement of a new prosthesis. Depending on the severity of infection and prognosis, this may consist of a one- or two-step process [11]. Unfortunately, multiple revisions deplete bone stock and cause soft tissue injury, diminishing the chance for successful subsequent THA [12].

Poor candidates for surgical revision are placed on an indefinite regimen of antibiotics to manage underlying infection and prevent complications. Culture of pathogen should direct the clinician's choice of antibiotic therapy. In addition, oral bioavailability, drug interactions, cost, and toxicity must also be considered. Regardless, long-term antibiotic therapy is associated with significant side effects to the patient, and compliance remains an issue (such was the case with the patient presented in this report). Despite these negative implications, AST is considered a reasonable treatment modality for patients unwilling to undergo surgical revision or who are unable to tolerate multiple stage revision due to health concerns [13]. Failed antibiotic therapy or repeated failed surgical revision can necessitate excision arthroscopy of the hip, arthrodesis, or possibly lower limb amputation [13].

The case presented describes a patient who, despite guidance from his physician, discontinued AST and experienced a full recovery of his draining sinus tract and underlying infection. The authors have yet to identify literature describing a similar resolution of draining sinus tracts or deep tissue infection after discontinuing AST. Despite the resolution of this patient's sinus tract, the authors do not advocate for discontinuing AST in patients with a draining sinus tract. However, in spite of the fact that the MSIS consensus statement suggests that a draining sinus is a sure sign of PJI and that the assumption is that the infection will not go away until explant, this case was different.

## Figures and Tables

**Table 1:** Risk factors associated with periprosthetic joint infection of the hip [4, 8–10].

Perioperative	Male gender
	Hispanic
	Diabetes mellitus
	Vitamin D deficiency
	Tobacco use
	Obesity
	Immunosuppressive therapy
	Depression
	Anemia
	Chronic kidney disease
	Dementia
	Cardiovascular disease
	Alcohol abuse
	Malnutrition
Preoperative	Simultaneous bilateral arthroscopy
	Longer surgery time
Postoperative	Longer hospital stay
	Allogenic blood transfusion
	Atrial fibrillation
	Myocardial infarction
	Urinary tract infection
	Bacteremia
